# Improved Functionality of the Vasculature during Conventionally Fractionated Radiation Therapy of Prostate Cancer

**DOI:** 10.1371/journal.pone.0084076

**Published:** 2013-12-31

**Authors:** Vincent A. Potiron, Rym Abderrahmani, Karen Clément-Colmou, Séverine Marionneau-Lambot, Thibauld Oullier, François Paris, Stéphane Supiot

**Affiliations:** 1 Inserm, UMR892, Nantes, France; 2 Université de Nantes, Nantes, France; 3 CNRS, UMR6299, Nantes, France; 4 Department of Radiation Oncology, Institut de Cancérologie de l’Ouest, Saint-Herblain, France; 5 Plate-forme in vivo, Cancéropôle Grand-Ouest, Nantes, France; Alexion Pharmaceuticals, United States of America

## Abstract

Although endothelial cell apoptosis participates in the tumor shrinkage after single high-dose radiotherapy, little is known regarding the vascular response after conventionally fractionated radiation therapy. Therefore, we evaluated hypoxia, perfusion and vascular microenvironment changes in an orthotopic prostate cancer model of conventionally fractionated radiation therapy at clinically relevant doses (2 Gy fractions, 5 fractions/week). First, conventionally fractionated radiation therapy decreased tumor cell proliferation and increased cell death with kinetics comparable to human prostate cancer radiotherapy. Secondly, the injection of Hoechst 33342 or fluorescent-dextrans showed an increased tumor perfusion within 14 days in irradiated tumors, which was correlated with a clear reduction of hypoxia. Improved perfusion and decreased hypoxia were not explained by increased blood vessel density, size or network morphology. However, a tumor vascular maturation defined by perivascular desmin+/SMA+ cells coverage was clearly observed along with an increase in endothelial, zonula occludens (ZO)-1 positive, intercellular junctions. Our results show that, in addition to tumor cell killing, vascular maturation plays an uncovered role in tumor reoxygenation during fractionated radiation therapy.

## Introduction

Although the sensitivity of tumors to radiation therapy (RT) is largely dependent on the intrinsic radioresistance of cancer stem cells [1], other data suggest that the sensitivity of the endothelium also plays an important role [2]. As a result of excessive production of angiogenic molecules, blood vessels in solid tumors display characteristic features such as dilated microvessels, incomplete endothelial lining, compression by tumor cells, excessive branching and highly irregular architecture. At a cellular level, an incomplete maturation of the capillaries is noted with absent or detached perivascular cells, absent or too thick basement membrane and lack of endothelial cells junction. This abnormal vasculature causes hypoxia that further impacts the efficacy of irradiation because 1) lack of oxygen reduces the amount of reactive oxygen species induced by irradiation and 2) hypoxia selects radioresistant mutant cells [3]. During fractionated radiotherapy, the proportion of hypoxic cells rapidly increases following the treatment fraction since normoxic cells are preferentially and rapidly killed by irradiation. In the next hours, because of reduced oxygen consumption by damaged cells, improved perfusion, and reduced cell density, hypoxic tumor cells may gain an easier access to oxygenation, and therefore become more sensitive to the subsequent fraction of irradiation. This process of reoxygenation is believed to confer a therapeutic advantage to CFRT through a progressive decrease of normoxic tumor cells and easier access of hypoxic cells to oxygen [3].

Blood vessels are largely affected by RT depending on the number of fractions, dose rate, total radiation dose and fraction size [4], [5]. Single high-dose irradiation can induce rapid apoptosis of the vasculature [6], [7]. In addition, both single high-dose and hypofractionated irradiation induce endothelial cell apoptosis, thereby decreasing vascular density [8], with surviving vessels more dilated [9], [10]. However, the importance of vascular damage in tumors receiving conventionally fractionated radiation therapy (CFRT: 1.8-2 Gy per fraction) is more controversial [11], [12]. In normal brain blood vessels, very recent information suggests that, conversely to larger doses, a 2 Gy-irradiation induces minimal endothelial cell apoptosis followed by a later increase in vessel diameter, microvascular density and vessel leakiness [13]. However, quiescent endothelial cells are more resistant to irradiation than proliferating endothelial cells [14], [15]. Therefore, the effects of CFRT on the tumor endothelium may differ from the normal endothelium. Moreover, to understand the effects of clinical radiotherapy, specific studies on tumor blood vessels have to be conducted in orthotopic models since the extent of hypoxia and vascularization is largely affected by the site of tumor engraftment [16], [17].

To study the effects of CFRT on the tumor vasculature and hypoxia, we selected a prostate cancer model, because CFRT up to total doses of 74–80 Gy plays a major role in localized prostate cancer patients [18], and because the response of prostate tumors to irradiation is highly sensitive to hypoxia [19], [20]. Moreover, prostate cancers display terminal growth arrest features as the dominant mode of radiation-induced cell death [21], [22], and this slow kinetic of cell kill following RT may largely impact tumor reoxygenation. We analyzed the evolution of tumor and vasculature structural and functional changes and their impact on the tumor microenvironment using a clinically-relevant fractionation scheme (2 Gy×5 fractions/week during 2 weeks) in an orthotopic prostate cancer model. Our results suggest that conversely to hypofractionated radiotherapy, CFRT does not affect vessel density, but remodels tumor vasculature by increasing perivascular coverage, improving vessel perfusion and leading to decreased hypoxia.

## Results

### Conventionally fractionated radiation therapy controls prostate tumor growth

**Figure 1:**
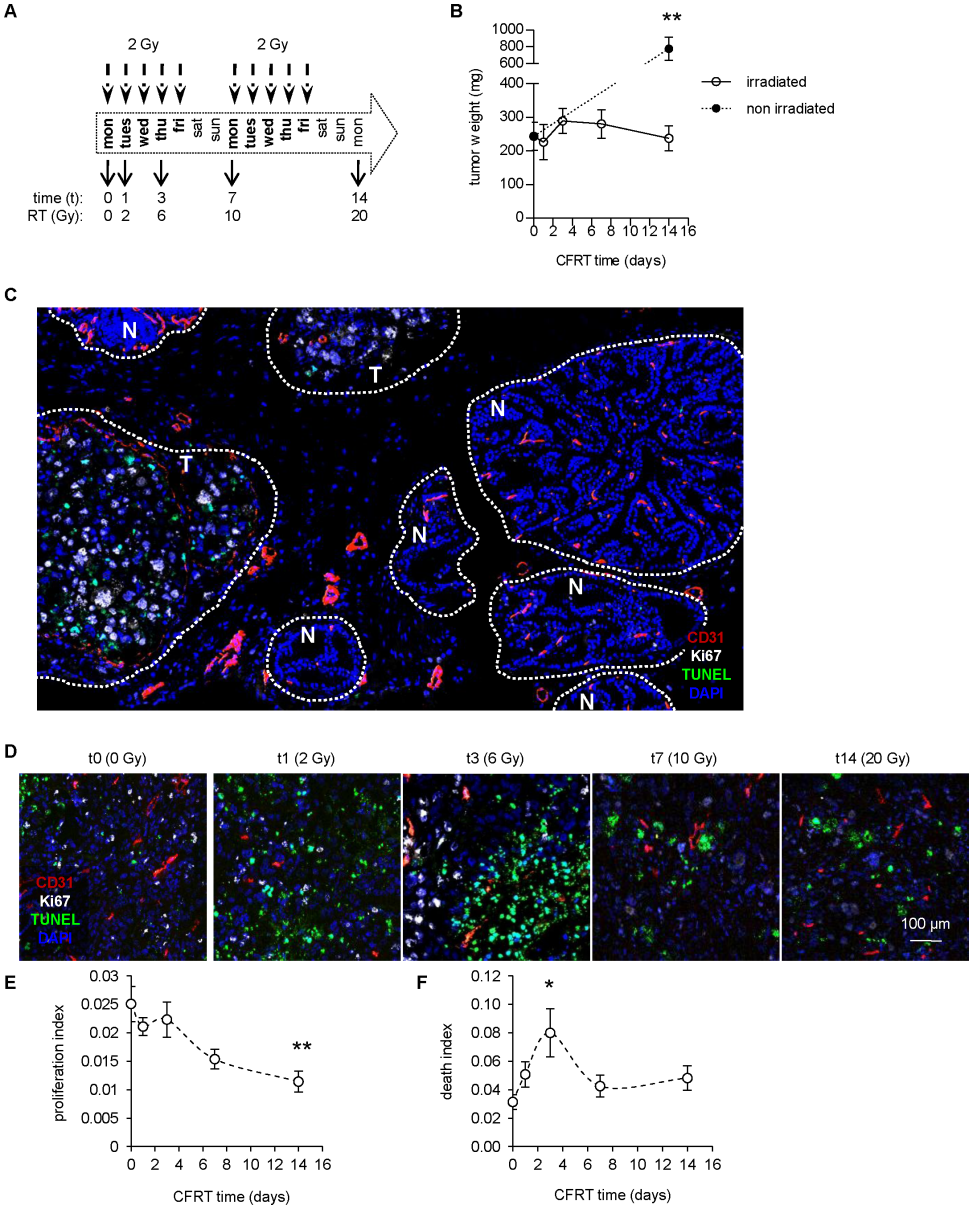
Control of prostate tumor growth by fractionated irradiation. (**A**) Scheme of the conventional fractionated radiation therapy (CFRT) protocol used for treatment of established prostate tumors. (**B**) Weight of dissected tumors after the indicated time of CFRT. Values  =  average of n≥6 ± sem. Statistical comparison vs. t14 irradiated. (**C**) Example of a tumor treated for one week, showing TUNEL+ tumor cells ("T") and normal adjacent tissues ("N") mainly TUNEL-. Tumor and normal tissues were identified with DAPI based on nuclei morphology, size, staining intensity and spatial organization of cells. (**D**) Pseudo-confocal images of tumors during CFRT, stained for Ki-67/TUNEL/CD31. (**E,F**) Image quantification of cell proliferation (Ki-67 index, **E**) and cell death (TUNEL index, **F**). Statistical comparisons vs. t0.

To study changes of the tumor and its microenvironment during a clinically relevant protocol of radiation therapy, we induced orthotopic prostate cancer in mice. Two weeks after cells were injected, tumor-bearing animals received a daily dose of 2 Gy irradiation centered on the lower abdomen, each day of the week for two weeks (conventionally fractionated radiation therapy, CFRT; Fig. 1A). This protocol mimics the first two weeks of radiation therapy for prostate cancer patients. Animals were sacrificed and analyzed at 0 (non-irradiated), 1 (2 Gy), 3 (6 Gy), 7 (10 Gy) and 14 days (20 Gy) during the treatment. As expected, irradiation led to a marked decrease in tumor weight compared to non-treated animals (irradiated: 237±37 mg vs. non-irradiated: 777±135 mg, p<0.01, Fig. 1B). Also, irradiation had a preferential effect on proliferating tumor cells, compared to adjacent healthy tissues (Fig. 1C). Reduction of cell proliferation was visible from day 1, and more pronounced with time (–54% at t14 vs. t0, p<0.01, Fig. 1D,E). In parallel, cell death increased rapidly at day 3 (+ 109%, p<0.05) and remained non-significantly higher than baseline thereafter (Fig. 1D,F). Overall, these results are in line with the slow-kinetic responses observed for prostate cancer patients.

### CFRT reduces hypoxia and increases tumor perfusion

**Figure 2:**
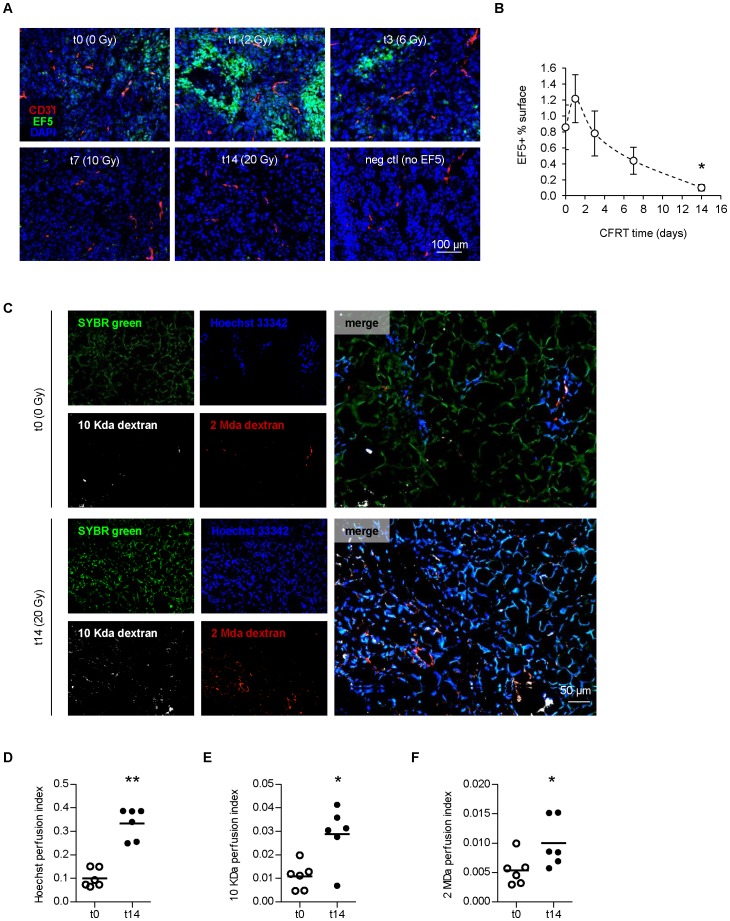
Fractionated irradiation reduces hypoxia and increases tumor perfusion. (**A**) Pseudo-confocal images of tumors during CFRT, stained for hypoxia (EF5) and endothelial cells (CD31). (**B**) Image quantification of EF5+ surface in tumors during CFRT. Values represent the average of n≥13 per point ± sem. (**C**) Pseudo-confocal images of tumors perfused with Hoechst 33342 and 10 kDa/2 MDa dextrans before (t0) or after 2 weeks of CFRT (t14). SYBR green was used as a counterstain of total cell nuclei. (**D,E,F**) Image quantification of Hoechst+ (**D**), and medium (**E**) and large (**F**) dextran+ surfaces in tumors during CFRT (n  =  6). (**B**,**D**,**E**,**F**). Statistical comparisons vs. t0.

Hypoxia results from oxygen overconsumption by tumor cells as well as tumor blood vessel abnormality. To determine the pattern of hypoxia, a major radioresistance factor, during CFRT, we used the extrinsic marker EF5 [23], which highlights severe (< 0.2% O_2_), radiobiologically-relevant hypoxia (Fig. S1A-C). EF5-positive surface was moderately increased at day 1 (+45%), although not significantly, with some hypoxic areas in the vicinity of blood vessels, suggesting that not all vessels were perfused (Fig. 2A-B). However, hypoxia decreased in all subsequent time points, to reach only 11.4% of t0 at 2 weeks (p<0.05, Fig. 2A-B).

To assess vessel functionality, fluorescent molecules of different sizes were injected intravenously. Large (2 MDa dextran) molecules that remain intravascular account for perfusion, medium (10 kDa dextran) for interstitial diffusion and small (Hoechst 33342, 616 Da) for permeability. While in non-irradiated tumors, only discrete areas were positively stained, the distribution surface of all molecules was largely increased after 2 weeks of CFRT and was more homogeneous (Fig. 2C-F). Distribution of Hoechst reached a value near that of the normal prostate (t0: 0.10 vs. t14: 0.33, p<0.01, Fig. 2C-D; normal: 0.41, Fig. S2). The overall surface of medium (10 KDa) and large (2 MDa) dextrans was also increased (t0: 0.011 vs. t14: 0.029, p<0.05, Fig. 2C,E and t0: 0.0054 vs. t14: 0.010, p<0.05, Fig. 2C,F), although not comparable to normal tissues (0.12 and 0.02, Fig. S2) as the microvessel density (MVD) is ≈ 3 times lower in tumors (Fig. S3A-C). Both dextrans remained punctuated, showing no sign of substantial diffusion that would represent vessel leakiness (2 MDa) or abnormal permeability (10 KDa). The similar increase regardless of molecule size is consistent with an increase in perfusion (e.g. increased blood flow). Interestingly, no significant increase in perfusion was observed in the normal prostate acini (Fig. S4A-D). Thus, CFRT preferentially improves vessel perfusion in the context of the tumor microenvironment.

### CFRT does not induce changes in vessel density, size or distribution

**Figure 3:**
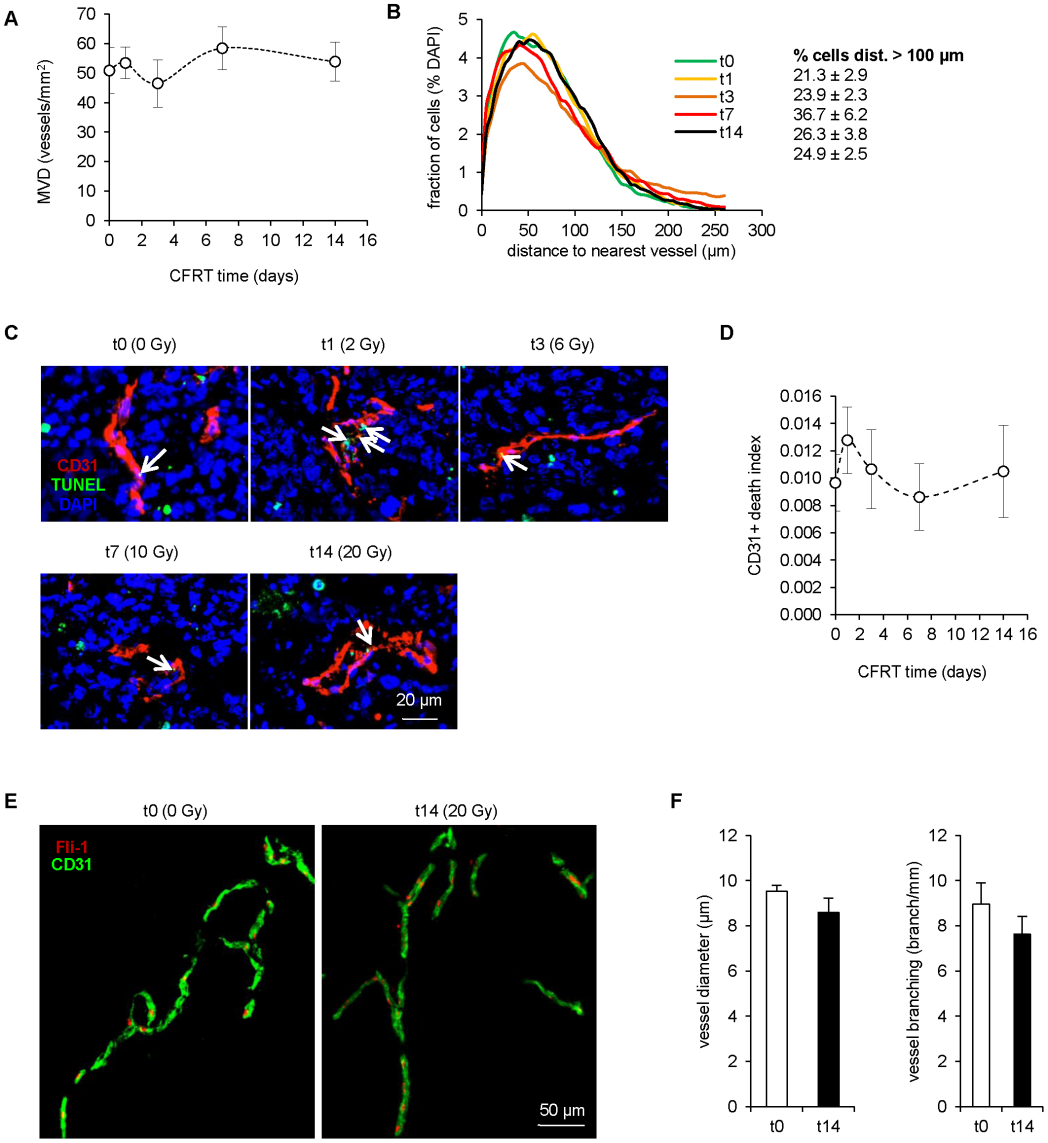
Maintenance of vascular density and distribution during fractionated irradiation. (**A**) Microvessel density in tumors during CFRT. Values represent the average of n≥13 per point ± sem. (**B**) Distance profile between cells and the closest blood vessel, from tumors during CFRT. Profiles are based on n≥13. Statistical comparisons vs. t0. (**C**) Pseudo-confocal images of tumor-associated blood vessels (CD31+) stained for TUNEL during CFRT. Arrows: TUNEL+/CD31+ cells. (**D**) Image quantification of CD31+/TUNEL+ surface. Values represent the average of n≥13 per point ± sem. (**E**) Representative Z-stack images of 100 µm-thick tumor sections before (t0) or after 2 weeks of CFRT (t14) and stained for blood vessels (CD31+/Fli-1+). (**F**) Image analysis of blood vessel network from 100 µm-thick tumor sections. Values represent the average of n  =  9 per point ± sem.

Improved perfusion in the context of tumor chemotherapy has been associated with vascular normalization, and potentiates radiation therapy [24]. However, changes of the tumor-associated vasculature during CFRT have not been studied. We therefore investigated microvessel density and structure in tumors irradiated at different times. MVD was stable during the time course of the experiment, from 51±8 microvessels/mm^2^ at t0 to 54±7 at t14 (Fig. 3A), unlike unirradiated tumors, which exhibited a significant increase (from 47±7 to 80±8, p<0.01, Fig. S5A,B). Of note, this is well below that observed in the normal prostate with a MVD of 166±6 (Fig. S3C, p<0.01 vs. tumor at t0), which may explain the hypoxia detected in untreated tumors. The maintenance of MVD prompted us to determine whether endothelial cell death occurs during CFRT. Therefore, we measured the TUNEL index in CD31+ cells. CFRT did not induce EC death (Fig. 3C,D), despite a tendency at day 1 (+28%, p  =  0.37) that was coherent with the non-significant MVD minimum at day 3 (Fig. 3A). Moreover, the amount of TUNEL+ staining was not consistent with massive endothelial cell damage. Next, to account for blood vessel distribution heterogeneity, we determined the distance of cells (identified by DAPI) to the closest vessel. Using this analysis, the slight and transient MVD reduction observed at day 3 (Fig. 3A) resulted in a mild but unsignificant shift of cells toward a theoretically hypoxic environment (> 100 µm from the closest vessel, Fig. 3B). However, at the time where hypoxia was absent and perfusion was augmented (t14), the distance profile of cells to blood vessels was identical to unirradiated tumors (t0: 21.3 vs. t14: 24.9% of cells at more than 100 µm). In addition, values were largely farther than those encountered in the normal prostate ( S3A,F). Thus, the increased delivery of Hoechst/dextrans observed after irradiation might be due to vessel quality rather than quantity. Therefore, we analyzed vessel network morphology by scanning tissues over a 100 µm thickness. Nevertheless, compared to unirradiated tumors, tumors harvested at day 14 during CFRT did not show any difference in vessel branching (t0: 8.9 branch/mm vs. t14: 7.6, p  =  0.32; Fig. 3E,F), diameter (t0: 9.5 µm vs. t14: 8.6 µm, p  =  0.21). Overall, these results indicate that the reduced hypoxia/augmented perfusion during CFRT does not correlate with better vascular density, topography or morphology.

### CFRT induces maturation of the vascular wall

**Figure 4:**
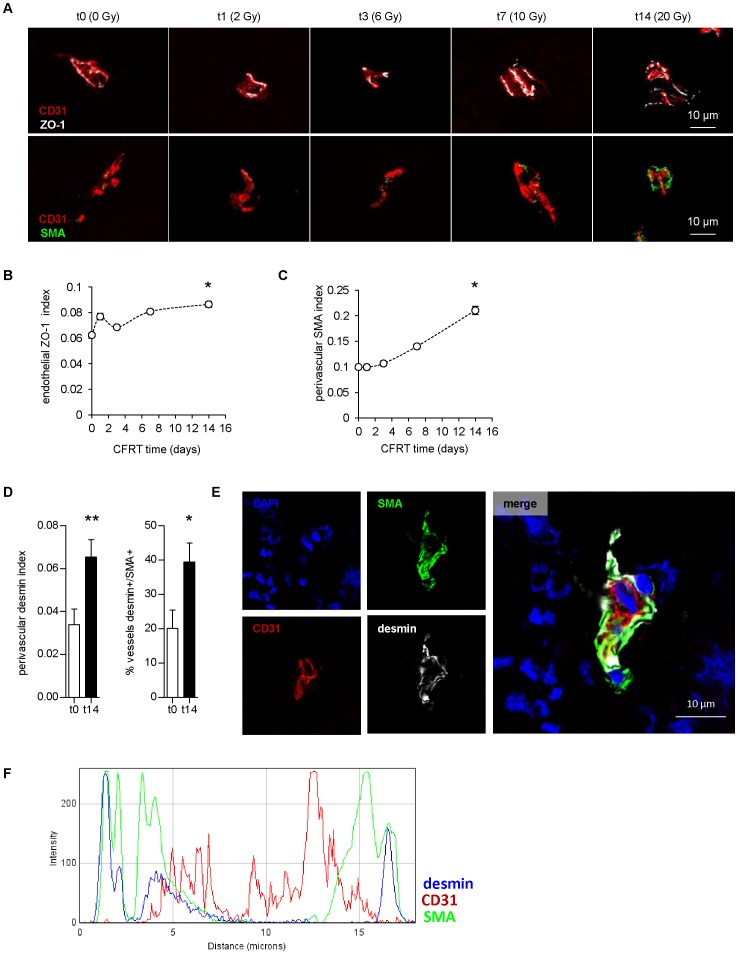
Fractionated irradiation induces vascular remodeling. (**A**) Pseudo-confocal images of tumor blood vessels during CFRT and stained for ZO-1/CD31 (top) or SMA/CD31 (bottom). (**B**,**C**). Image quantification of ZO-1+/CD31+ (**B**) and peri-CD31+ SMA surfaces (**C**). Values represent the average of n≥13 per point ± sem. (**D**) Image quantification of peri-CD31+ desmin surface and frequency of desmin+/SMA+ vessels. (**B**,**E**,**D**) Statistical comparisons vs. t0. (**E**) Representative confocal images of a blood vessel from a 14-day treated tumor stained for CD31/desmin/SMA. (**F**) histogram analysis of CD31/desmin/SMA pseudocolor profile of confocal image cross-section.

Abnormal tumor vessels often exhibit non-continuous endothelial wall, resulting in exacerbated permeability and leakiness. To appreciate endothelial wall cohesion, we stained for zonula-occludens (ZO)-1, a protein involved in tight junctions. Endothelial (CD31+)-specific ZO-1 index was significantly upregulated after 2 weeks of CFRT (+38% of t0, p<0.05; Fig. 4A,B). More importantly, this was associated with increased coverage of endothelial cells by alpha smooth-muscle actin (SMA) positive cells (+111% of t0, p<0.05; Fig. 4A,C). By confocal microscopy and histogram analysis, ZO-1 was confirmed to be endothelial (inside CD31-expressing cells) and continuously expressed, specifically after irradiation (Fig. S6A-B). In opposite, SMA was non-endothelial and juxtaposed to the endothelial wall. To precise the nature of the perivascular cells, tissues were co-stained for desmin, a pericyte marker [25]. In addition to the SMA index, desmin+ perivascular cells were also increased (t0: 0.034 vs. t14: 0.065, p<0.01; Fig. 4D). Moreover, SMA and desmin were co-expressed by the same cells (Fig. 4E,F). Also, the frequency of desmin+/SMA+ covered vessels doubled between day 0 and day 14 of CFRT (t0: 20.0 vs. t14: 39.4, p<0.05). Of note, SMA and desmin were also co-expressed in the rare covered non-irradiated vessels (Fig. S7). In the normal prostate, desmin was expressed by both micro (intra-acini) and macro (inter-acini) -vessels, although SMA was strictly restricted to macro-vessels (Fig.S3A,D-E, Fig. S8A-B). Moreover, no upregulation of SMA was detected in irradiated normal microvessels (Fig. S9A,C), nor in non-irradiated tumor microvessels (Fig. S5C,D). Thus, the increase of perivascular desmin+/SMA+ cells is specific of irradiated tumor microvessels.

## Discussion

Although several studies suggest that irradiation leads to vessel destruction, the effect of conventionally fractionated irradiation has been largely unexplored. We used an orthotopic model of prostate cancer with a clinically-relevant fractionation scheme, inducing modest cell death, increasing proliferation arrest with dose and differential effect between normal/tumor cells. We find that CFRT nearly abrogated tumor hypoxia after 2 weeks of treatment, together with an increased perfusion. Unexpectedly, these effects were not associated with significant changes in vessel morphology or density but rather with phenotypic maturation of the vascular wall, including endothelial cells (CD31+/ZO-1+) and pericytes (desmin+/SMA+). Overall, these observations indicate that vascular adaptation plays an uncovered role in tumor reoxygenation during fractionated radiation therapy.

We did not observe a drastic increase in apoptotic endothelial cells 24h after daily 2 Gy irradiations, and MVD remained stable, consistent with recent information in normal brain blood vessels [13]. These results are contrasting with previous studies using higher dose irradiation, where endothelial cell death and decline in vessel density were reported, although at earlier time points [2], [4]. Particularly, fractions of 4 and 12 Gy for 2–3 weeks led to increased endothelial cell death and decreased MVD in subcutaneous tumors [8], [9], [10]. This is surprising since endothelial apoptosis is thought to occur only at single doses ≥ 10–15 Gy [2], [4]. However, EC death might depend on the tumor setting, since only minor vascular changes were noted 24h after a single dose of 15 Gy in another prostate cancer model [26]. Different endothelial cell states between studies may account for differences in radiosensitivity, since quiescent endothelial cells are more resistant to DNA damage induced by irradiation, but not to short-term membrane-induced cell death [14] [15]. This may explain that the observed increases in pericytic coverage and perfusion were only significant in the irradiated tumor compartment, where the microvascular environment may be more responsive/permissive. These results illustrate the complexity of the tumor vasculature and its heterogeneous response to treatments. Perhaps the importance of an orthotopic setting is often under-appreciated since blood vessels display strong heterogeneity between organs (e.g. androgen receptor expression for the prostate endothelium, the blood-brain barrier etc..). Indeed, PC3-derived tumors grown subcutaneously have substantially greater vessel size and density (≈ x3, data not shown). Most importantly, recent studies using higher fraction doses are in agreement with our work for a decrease in tumor hypoxia and increased perfusion together with pericyte coverage [9], [10], except one report using very high (30 Gy) single dose irradiation [27]. Collectively, these studies suggest that increased perivascular coverage might play an essential role in vessel perfusion and reoxygenation after fractionated irradiation.

Preserving the vasculature is now viewed positively for cancer treatment, owing to the concept of vascular normalization. The concept beyond this observation was that destroying the vasculature would produce hypoxia that renders irradiation less effective. The initial observation was that inhibition of overproductive angiogenesis with an anti-VEGFR2 antibody temporarily recruits pericytes and downregulates tumor hypoxia [24]. Since uncontrolled EC sprouting has been associated with non-functional angiogenesis, irradiation-induced pruning of vascular sprouts may result in decreased network complexity and improved perfusion [15]. However, we did not find key features that characterize normalized blood vessels since branching, MVD, tortuosity and vessel diameter were unchanged. Although perfusion was similar in some aspects to that of the normal prostate, vessel phenotype was distinct, with higher ZO-1 in irradiated vessels than what is normally encountered. In addition, in the normal prostate, the perimeter of micro-vessels was consistently desmin+/SMA–, whereas SMA expression was strictly restricted to large, inter-acini vessels. This vascular hierarchy is also found in human samples [28]. In contrast, irradiated tumor micro-vessels (≈ 10 µm diameter) gained perivascular SMA expression. These data indicate that the vessel wall might differentiate uniquely following irradiation, consistent with other reports on perivascular cell response to inflammatory stress (TGFb, PDGFb).

Aberrant expression of endothelial junctional proteins, including ZO-1, can occur in absence of endothelial cell polarization [29] or shortly (e.g. min.) after irradiation [30]. However, the increased endothelial ZO-1 observed here after 2 weeks is likely related to vessel stabilization since 1) our perfusion data suggest that most vessels are efficiently lumenized and 2) the pattern of ZO-1 is membranous, linear and continuous. As for the perivascular maturation, the close association with CD31-counterstained endothelial cells and the presence of two markers (desmin/SMA) identify the surrounding cells as pericytes [25]. Perivascular cells are important regulators of vascular formation, stabilization, remodeling and function to generate a mature, quiescent vasculature [31]. Maturation of vessels occurs through reciprocal paracrine signaling where EC-derived factors attract pericytes and the later favor the establishment of endothelial cell junctions [32]. Of importance, pericyte-secreted cues protect ECs from radiation-induced death *in vitro* [33]. This suggests that vessels without pericyte coverage would be more vulnerable to irradiation, as hypothesized recently by Chen et al [9]. Therefore, fractionated irradiation regimens, which allow for vascular maturation, may improve tumor vascular function.

Our data indicate a reduction of the hypoxic fraction after two weeks of treatment and are in agreement with recent data in subcutaneous tumors [9], [10], [34]. Tumor reoxygenation was defined as the concept that chronically hypoxic tumor cells gain a better access to oxygen during fractionated radiation therapy because aerobic tumor cells are eliminated by previous fractions [35]. In our model, tumor reoxygenation happened not only in the context of decreased tumor cell proliferation and increased cell death, but also with an improved functionality of the vasculature evidenced by perfusion and diffusion of small molecules. This means that tumor reoxygenation is not only due to increased killing of tumor cells and less oxygen consumption, but also to a better distribution of oxygen by blood vessels. In different experimental tumor models as well as in clinical studies, intertumoral heterogeneity in the kinetics of oxygenation during fractionated was shown [36], [37], [38], [39]. For example, in squamous cell carcinomas, hypoxia decreased during CFRT in a majority of tumor lines, whereas in some tumor cell lines a temporal increase or no change was observed and fraction of perfused vessels and vascular area showed only modest changes [34]. Furthermore, our results suggest that during CFRT, stabilization of vessels is necessary so that the 2^nd^ week fractions become more efficient. While there is increasing interest in high-dose hypofractionated protocols, especially in prostate cancer patients [40], these data raise the question whether such protocols preserve the advantage of increasing oxygen levels and thereby the therapeutic index [41]. In other tumor models however, hypofractionated (3 Gy/fraction radiotherapy) improved oxygenation only during the early phase of the treatment, but led to a considerable decrease of tumor oxygenation in the later phase of irradiation (> 45 Gy) [42]. Thus, more knowledge is needed regarding the influence of vascular maturation on the radiosensitivity of tumor cells.

In summary, we found that conventionally fractionated irradiation induces vascular maturation, along with increased perfusion/decreased hypoxia. These results imply that the vascular microenvironment plays a role in tumor reoxygenation, in addition to the known effects on tumor cells. It is unclear whether hypofractionated protocols may allow vascular maturation or instead lead to vascular sterilization and hypoxia. The impact of such protocols on tumor blood vessels and on the clinical outcome should be the prospect of further studies.

## Materials and Methods

### Orthotopic prostate tumorigenesis and tissue collection

Seven-week old male NMRI-nude mice (Janvier, Saint Berthevin, France) were anesthetized using ketamine/xylazine (50/15 mg/kg) and 2×10^6^ PC3-luc cells (Caliper Life Sciences, Villepinte, France) were injected in the left dorsolateral lobe of the prostate by surgery. After two weeks, tumor uptake was verified by luminescence using a PhotonImager (BiospaceLab, Paris, France) and groups with similar tumor bioluminescence signal were formed. Experiments began 3 days later. When appropriate, animals were sacrificed, tissues were excised and weighted if necessary, and frozen (–80°C) embedded in OCT medium (Sakura Finetek, Villeneuve D'ascq, France). Samples were collected at the time of the next CFRT fraction, to analyze tumor state at the time where it should be treated.

All animal experiments were carried out in accordance with the European Council Directive 2010/63/UE and approved by the local Animal Care and Use Committee (Comité d’Ethique en Expérimentation Animale des Pays-de-la Loire, C2EA-06).

### *In vivo* hypoxia and perfusion

Hypoxia and perfusion were determined using specific markers that were administered i.v. in saline buffer. For hypoxia, 300 µl of 3 mg/ml EF5 (hypoxia-imaging.org) were injected 2h before sacrificing [23], [43] whereas 300 µl of mixed perfusion markers (1.6 mg/ml Hoechst 33342, 0.6 mg/ml Alexa^647^-10 kDa dextran and 3 mg/ml rhodamine-2 MDa dextran; Life Technologies, Saint Aubin, France) were injected 3 min ahead of tissue collection [44] Tissues were snap-frozen, cryosected, counterstained with SYBR Green (Fisher Scientific, Illkirch, France) for total cell nuclei and mounted in Prolong Gold without DAPI (Life Technologies).

### Radiation therapy

Irradiations were performed using a CP-160 X-ray irradiator (Faxitron, Lincolnshire, IL) with a 0.3 mm Cu filter, an accelerating voltage of 160 kV and a dose rate of 1.3 Gy/min. Animals received a dose (fraction) of 2 Gy daily monday-friday for two weeks, centered on a lower abdomen field of 2 cm×2 cm using lead shields designed to minimize irradiation to normal tissues and bones (hips). This scheme (defined here as conventionally fractionated radiation therapy, CFRT) is standard clinical practice for prostate cancer.

### Immunohistochemistry

Tissues were cryosected at 5 µm thickness (except for network analysis: 100 µm), fixed with 4% paraformaldehyde and permeabilized with 0.05% Triton-X100. IHC was done using standard staining and washing procedures [45]. For multiple staining, antibodies were incubated sequentially: the first, overnight followed by its secondary and fixing, then CD31 as the second and SMA as the third. The following primary antibodies were used: rat anti-mouse CD31 (BD Biosciences, Le Pont-de-Claix, France), rabbit anti-ZO-1 (Life Technologies), rabbit anti-desmin (Ozyme, St-Quentin-en-Yvelines, France), rabbit anti-Ki67 (Dako, Les Ulis, France), rabbit anti-Fli1 (Fisher Scientific) Alexa488-conjugated mouse anti-EF5, Cy3-conjugated mouse anti-alpha smooth muscle actin (Sigma, Saint-Quentin Fallavier, France). The secondary antibodies were: Alexa^647^-conjugated goat anti-rabbit, Alexa^488^-conjugated goat anti-rat (Life Technologies). TUNEL was performed as recommended by the manufacturer (Roche, Boulogne-Billancourt, France) as the last staining step. Slides were mounted in Prolong Gold with DAPI (Life Technologies) for nuclei counterstaining (except for perfusion assay).

Slides were observed under an Axiovert 200M ApoTome ("pseudo-confocal") microscope, with Axiovision 4.8 software (Carl Zeiss, Le Pecq, France). Mosaics of fields up to 4 mm^2^ were recorded at the 63x oil objective, and four Z-steps of 1 µm were stacked into one image. Slices of 100 µm thickness were usually recorded in ≈ 60 Z-steps of 1 µm, owing to tissue flattening after dehydration. Confocal microscopy was done with a A1R (Nikon Instruments, Champigny-sur-Marne, France) with Nis-Elements software, a numerical aperture of 1 and slice scanning averaging of 4.

### Image analysis

Analyses were done on uncompressed original 16-bit images of high resolution mosaics (pseudo-confocal), except for color distribution along vessel cross-sections (confocal; "histogram analysis", see later). Two types of values were recorded: values for surface-based analyses correspond to staining surfaces (µm^2^) considered positive for the staining of interest (ex, CD31 area) divided by the surface of the region of interest (ROI; ex, total tissue); values for object-based analyses correspond to the absolute number of elements considered positive (ex, number of vessels) divided by the surface of the ROI (ex, total tissue).

Surface-based analyses were performed using ImageJ 1.46r software (National Institutes of Health, USA). Object-based analyses were done using Volocity 6.1.1 (Perkin Elmer, Courtaboeuf, France). Segmentation (determination of positive areas/elements) was based on pixel intensity ratio to the neighboring background and to a negative control. In all cases, appropriate automatic identification of positive areas/elements was verified by the experimenter to avoid analyzing microscopic/staining artifacts.

For tumor-related analysis, remaining normal nodules (acini) and their contractile desmin+/SMA+ septa were manually cropped from the image, and conversely for normal tissues. Three ROIs were defined: total tissue ROI (TT) was defined as regions positive for DAPI staining and their surrounding 10 µm to exclude luminal and acellular surfaces from the analysis; vascular ROI (VS) was defined as the CD31 areas; perivascular ROI (PVS) was defined as the 5 µm areas lining VS.

For proliferation, death and EF5 indexes, values are Ki67+, TUNEL+ and EF5+ surfaces divided by TT. Endothelial-specific TUNEL and ZO-1 indexes are CD31+(VS)/TUNEL+ and CD31+(VS)/ZO-1+ surfaces divided by VS. Perivascular SMA and desmin indexes are PVS/SMA+ or PVS/desmin+ divided by VS. For histogram analysis (Fig. 4F, S6 and S7), raw 16-bit images corresponding to a single confocal z-plane were converted to a 8-bit RGB colorspace. Pixel values should therefore not be considered as quantitative. For cells to vessel distance profile analysis, an original selection was created as the border of VS ROIs. DAPI+ surface was determined in the selection and all the subsequent selections made by enlarging by 5 µm. Percent of total DAPI+ surface was calculated for each 5 µm gap, and plotted as the distance to blood vessel (smoothed profile). For vessel network analysis, segmented VS ROIs over 100 µm-thick sections were skeletonized and detail-analyzed using the "skeleton" plugin of ImageJ.

### Statistical analysis

Statistical analyses were performed by Two-tailed Mann-Whitney test with 95% confidence estimations, or with Kruskal-Wallis test with alpha risk  =  0.05 followed by Dunn's post-test (GraphPad Prism5, La Jolla, California, USA). Data were considered significant when p<0.05 (*), <0.01 (**) and <0.001(***).

## Supporting Information

Figure S1 **Oxygen sensitivity of EF5 in PC3 cells.** (**A**) Images of cells cultured as indicated and exposed to EF5 for 2h and stained using anti-EF5 (top) or competed antibody (bottom). (**B**) Image quantification of EF5 staining observed in (**A**). Values represent the average of n  =  3 per point ± sem and are normalized to 21% O_2_ (value  =  100). (**C**) Pseudo-confocal images of tumors injected (middle and right) or not (left) with EF5 and stained using anti-EF5 (left, right) or competed antibody (middle). Graph: EF5+ surface in uninjected ("neg") and injected ("pos") hypoxic tumor.
(TIF)

Figure S2 **Perfusion of the normal prostate.** Pseudo-confocal images of the normal prostate of untreated mouse injected with Hoechst 33342 and 10 kDa/2 MDa dextrans. SYBR green was used as a counterstain of total cell nuclei.
(TIF)

Figure S3 **Normal and tumor-associated vasculature of the prostate.** (**A**) Pseudo-confocal images of the normal prostate of untreated mouse stained for CD31/ZO-1/α-SMA. (**B**) Pseudo-confocal images of untreated mouse prostate tumor stained for CD31/ZO-1/α-SMA. (**C,D,E**) Quantifications in untreated normal mouse prostate ("normal") and untreated mouse prostate tumor ("tumor"). (**F**) Distance profile between cells and the closest blood vessel in untreated normal and tumor mouse prostate. Profiles are based on n≥6. (**C**,**D**,**E**,**F**). Statistical comparisons vs. normal.
(TIF)

Figure S4 **Fractionated irradiation does not increase perfusion of normal prostate acini.** (**A**) Pseudo-confocal images of normal prostate acini perfused with Hoechst 33342 and 10 kDa/2 MDa dextrans before (t0) or after 2 weeks of CFRT (t14). SYBR green was used as a counterstain of total cell nuclei. (**B,C,D**) Image quantification of Hoechst+ (**B**), and medium (**C**) and large (**D**) dextran+ surfaces in normal prostate acini during CFRT (n  =  6). Statistical comparisons vs. t0.
(TIF)

Figure S5 **Non-irradiated tumors exhibit increased MVD but not vascular maturation.** (**A, B**) Microvessel density in sham-irradiated (0 Gy) tumors. (**A**) Pseudo-confocal images. (**B**) Quantification; values represent the average of n≥13 per point ± sem. (**C**) Pseudo-confocal images of non-irradiated tumor blood vessels stained for SMA/CD31. (**D**) Image quantification of peri-CD31+ α-SMA surface. Values represent the average of n≥13 per point ± sem.
(TIF)

Figure S6 **Endothelial distribution of ZO-1 and perivascular distribution of SMA.** (**A,B**) Top: Representative confocal images of a blood vessel from an untreated (t0, **A**) or a 2-week treated (t14, **B**) tumor stained for CD31/ZO-1/SMA. Bottom: Histogram analysis of CD31/ZO-1/SMA pseudocolor profile of confocal image cross-section from (**A** or **B**).
(TIF)

Figure S7 **Perivascular co-expression of desmin and SMA.** Top: Representative confocal images of a blood vessel from an untreated (t0) tumor stained for CD31/desmin/SMA. Bottom: Histogram analysis of CD31/desmin/SMA pseudocolor profile of confocal image cross-section.
(TIF)

Figure S8 **Co-expression of desmin and SMA in the normal prostate.** (**A**,**B**). Representative confocal images of a blood vessel from an untreated normal mouse prostate stained for CD31/desmin/SMA. (**A**) intra- and (**B**) inter-acinus region.
(TIF)

Figure S9 **Irradiated microvessels of normal prostate acini exhibit no significant changes in MVD or vascular maturation.** (**A**) Pseudo-confocal images of normal blood vessels stained for SMA/CD31 during CFRT. (**B**) Microvessel density of normal prostate acini during CFRT. Values represent the average of n≥13 per point ± sem. (**C**) Image quantification of peri-CD31+ SMA surface. Values represent the average of n≥13 per point ± sem.
(TIF)

## References

[1] Baumann M, Krause M, Hill R (2008) Exploring the role of cancer stem cells in radioresistance. Nat Rev Cancer 8: 545–554. 18511937 10.1038/nrc2419

[2] Garcia-Barros M, Paris F, Cordon-Cardo C, Lyden D, Rafii S, et al (2003) Tumor response to radiotherapy regulated by endothelial cell apoptosis. Science 300: 1155–1159. 12750523 10.1126/science.1082504

[3] Bristow RG, Hill RP (2008) Hypoxia and metabolism. Hypoxia, DNA repair and genetic instability. Nat Rev Cancer 8: 180–192. 18273037 10.1038/nrc2344

[4] Park HJ, Griffin RJ, Hui S, Levitt SH, Song CW (2012) Radiation-induced vascular damage in tumors: implications of vascular damage in ablative hypofractionated radiotherapy (SBRT and SRS). Radiat Res 177: 311–327. 22229487 10.1667/rr2773.1

[5] Supiot S, Paris F (2012) [Radiobiology dedicated to endothelium]. Cancer Radiother 16: 11–15. 22326117 10.1016/j.canrad.2011.10.006

[6] Paris F, Fuks Z, Kang A, Capodieci P, Juan G, et al (2001) Endothelial apoptosis as the primary lesion initiating intestinal radiation damage in mice. Science (New York, NY) 293: 293–297. 10.1126/science.1060191 11452123

[7] Corre I, Niaudet C, Paris F (2010) Plasma membrane signaling induced by ionizing radiation. Mutat Res 704: 61–67. 20117234 10.1016/j.mrrev.2010.01.014

[8] Chen FH, Chiang CS, Wang CC, Tsai CS, Jung SM, et al (2009) Radiotherapy decreases vascular density and causes hypoxia with macrophage aggregation in TRAMP-C1 prostate tumors. Clin Cancer Res 15: 1721–1729. 19240176 10.1158/1078-0432.CCR-08-1471 PMC2868361

[9] Chen FH, Fu SY, Yang YC, Wang CC, Chiang CS, et al.. (2013) Combination of Vessel-Targeting Agents and Fractionated Radiation Therapy: The Role of the SDF-1/CXCR4 Pathway. Int J Radiat Oncol Biol Phys. 10.1016/j.ijrobp.2013.02.036 23601898

[10] Lan J, Wan XL, Deng L, Xue JX, Wang LS, et al (2013) Ablative hypofractionated radiotherapy normalizes tumor vasculature in lewis lung carcinoma mice model. Radiat Res 179: 458–464. 23480563 10.1667/RR3116.1

[11] Moeller BJ, Dreher MR, Rabbani ZN, Schroeder T, Cao Y, et al (2005) Pleiotropic effects of HIF-1 blockade on tumor radiosensitivity. Cancer Cell 8: 99–110. 16098463 10.1016/j.ccr.2005.06.016

[12] Fuks Z, Kolesnick R (2005) Engaging the vascular component of the tumor response. Cancer Cell 8: 89–91. 16098459 10.1016/j.ccr.2005.07.014

[13] Burrell K, Hill RP, Zadeh G (2012) High-resolution in-vivo analysis of normal brain response to cranial irradiation. PLoS One 7: e38366. 22675549 10.1371/journal.pone.0038366 PMC3366930

[14] Bonnaud S, Niaudet C, Pottier G, Gaugler MH, Millour J, et al (2007) Sphingosine-1-phosphate protects proliferating endothelial cells from ceramide-induced apoptosis but not from DNA damage-induced mitotic death. Cancer Res 67: 1803–1811. 17308123 10.1158/0008-5472.CAN-06-2802

[15] Imaizumi N, Monnier Y, Hegi M, Mirimanoff RO, Ruegg C (2010) Radiotherapy suppresses angiogenesis in mice through TGF-betaRI/ALK5-dependent inhibition of endothelial cell sprouting. PLoS One 5: e11084. 20552031 10.1371/journal.pone.0011084 PMC2884035

[16] Graves EE, Vilalta M, Cecic IK, Erler JT, Tran PT, et al (2010) Hypoxia in models of lung cancer: implications for targeted therapeutics. Clin Cancer Res 16: 4843–4852. 20858837 10.1158/1078-0432.CCR-10-1206 PMC2948600

[17] Penet MF, Pathak AP, Raman V, Ballesteros P, Artemov D, et al (2009) Noninvasive multiparametric imaging of metastasis-permissive microenvironments in a human prostate cancer xenograft. Cancer Res 69: 8822–8829. 19861534 10.1158/0008-5472.CAN-09-1782 PMC2783669

[18] Martin JM, Supiot S, Berthold DR (2011) Pharmacotherapeutic management of locally advanced prostate cancer: current status. Drugs 71: 1019–1041. 21668040 10.2165/11591500-000000000-00000

[19] Stewart GD, Ross JA, McLaren DB, Parker CC, Habib FK, et al (2010) The relevance of a hypoxic tumour microenvironment in prostate cancer. BJU Int 105: 8–13. 10.1111/j.1464-410X.2009.08921.x 19889065

[20] Milosevic M, Warde P, Menard C, Chung P, Toi A, et al (2012) Tumor hypoxia predicts biochemical failure following radiotherapy for clinically localized prostate cancer. Clin Cancer Res 18: 2108–2114. 22465832 10.1158/1078-0432.CCR-11-2711

[21] Bromfield GP, Meng A, Warde P, Bristow RG (2003) Cell death in irradiated prostate epithelial cells: role of apoptotic and clonogenic cell kill. Prostate Cancer Prostatic Dis 6: 73–85. 12664070 10.1038/sj.pcan.4500628

[22] Supiot S, Shubbar S, Fleshner N, Warde P, Hersey K, et al (2008) A phase I trial of pre-operative radiotherapy for prostate cancer: clinical and translational studies. Radiother Oncol 88: 53–60. 18423916 10.1016/j.radonc.2008.03.019

[23] Lord EM, Harwell L, Koch CJ (1993) Detection of hypoxic cells by monoclonal antibody recognizing 2-nitroimidazole adducts. Cancer Res 53: 5721–5726. 8242628

[24] Winkler F, Kozin SV, Tong RT, Chae SS, Booth MF, et al (2004) Kinetics of vascular normalization by VEGFR2 blockade governs brain tumor response to radiation: role of oxygenation, angiopoietin-1, and matrix metalloproteinases. Cancer Cell 6: 553–563. 15607960 10.1016/j.ccr.2004.10.011

[25] Armulik A, Genove G, Betsholtz C (2011) Pericytes: developmental, physiological, and pathological perspectives, problems, and promises. Dev Cell 21: 193–215. 21839917 10.1016/j.devcel.2011.07.001

[26] Roe K, Mikalsen LT, van der Kogel AJ, Bussink J, Lyng H, et al (2012) Vascular responses to radiotherapy and androgen-deprivation therapy in experimental prostate cancer. Radiat Oncol 7: 75. 22621752 10.1186/1748-717X-7-75 PMC3441216

[27] Maeda A, Leung MK, Conroy L, Chen Y, Bu J, et al (2012) In vivo optical imaging of tumor and microvascular response to ionizing radiation. PLoS One 7: e42133. 22927920 10.1371/journal.pone.0042133 PMC3425534

[28] Killingsworth MC, Wu X (2011) Vascular pericyte density and angiogenesis associated with adenocarcinoma of the prostate. Pathobiology 78: 24–34. 21474973 10.1159/000322739

[29] Zovein AC, Luque A, Turlo KA, Hofmann JJ, Yee KM, et al (2010) Beta1 integrin establishes endothelial cell polarity and arteriolar lumen formation via a Par3-dependent mechanism. Dev Cell 18: 39–51. 20152176 10.1016/j.devcel.2009.12.006 PMC3178410

[30] Gabrys D, Greco O, Patel G, Prise KM, Tozer GM, et al (2007) Radiation effects on the cytoskeleton of endothelial cells and endothelial monolayer permeability. Int J Radiat Oncol Biol Phys 69: 1553–1562. 17920784 10.1016/j.ijrobp.2007.08.039

[31] Bergers G, Song S (2005) The role of pericytes in blood-vessel formation and maintenance. Neuro Oncol 7: 452–464. 16212810 10.1215/S1152851705000232 PMC1871727

[32] De Bock K, Cauwenberghs S, Carmeliet P (2011) Vessel abnormalization: another hallmark of cancer? Molecular mechanisms and therapeutic implications. Curr Opin Genet Dev 21: 73–79. 21106363 10.1016/j.gde.2010.10.008

[33] Kwak HJ, Lee SJ, Lee YH, Ryu CH, Koh KN, et al (2000) Angiopoietin-1 inhibits irradiation- and mannitol-induced apoptosis in endothelial cells. Circulation 101: 2317–2324. 10811601 10.1161/01.cir.101.19.2317

[34] Yaromina A, Kroeber T, Meinzer A, Boeke S, Thames H, et al (2011) Exploratory study of the prognostic value of microenvironmental parameters during fractionated irradiation in human squamous cell carcinoma xenografts. Int J Radiat Oncol Biol Phys 80: 1205–1213. 21489709 10.1016/j.ijrobp.2011.02.015

[35] Van Putten LM, Kallman RF (1968) Oxygenation status of a transplantable tumor during fractionated radiation therapy. J Natl Cancer Inst 40: 441–451. 5644200

[36] Brurberg KG, Skogmo HK, Graff BA, Olsen DR, Rofstad EK (2005) Fluctuations in pO2 in poorly and well-oxygenated spontaneous canine tumors before and during fractionated radiation therapy. Radiother Oncol 77: 220–226. 16257074 10.1016/j.radonc.2005.09.009

[37] Baumann M, Appold S, Zimmer J, Scharf M, Beuthien-Baumann B, et al (2001) Radiobiological hypoxia, oxygen tension, interstitial fluid pressure and relative viable tumour area in two human squamous cell carcinomas in nude mice during fractionated radiotherapy. Acta Oncol 40: 519–528. 11504313 10.1080/028418601750288262

[38] Lyng H, Sundfor K, Rofstad EK (2000) Changes in tumor oxygen tension during radiotherapy of uterine cervical cancer: relationships to changes in vascular density, cell density, and frequency of mitosis and apoptosis. Int J Radiat Oncol Biol Phys 46: 935–946. 10705016 10.1016/s0360-3016(99)00497-6

[39] Brizel DM, Dodge RK, Clough RW, Dewhirst MW (1999) Oxygenation of head and neck cancer: changes during radiotherapy and impact on treatment outcome. Radiother Oncol 53: 113–117. 10665787 10.1016/s0167-8140(99)00102-4

[40] Supiot S, Crehange G, Latorzeff I, Pommier P, Paumier A, et al (2013) [Hypofractionated radiotherapy in prostate cancer]. Cancer Radiother 17: 349–354. 23973460 10.1016/j.canrad.2013.05.005

[41] Carlson DJ, Keall PJ, Loo BW, Jr, Chen ZJ, Brown JM (2011) Hypofractionation results in reduced tumor cell kill compared to conventional fractionation for tumors with regions of hypoxia. Int J Radiat Oncol Biol Phys 79: 1188–1195. 21183291 10.1016/j.ijrobp.2010.10.007 PMC3053128

[42] Zywietz F, Reeker W, Kochs E (1995) Tumor oxygenation in a transplanted rat rhabdomyosarcoma during fractionated irradiation. Int J Radiat Oncol Biol Phys 32: 1391–1400. 7635779 10.1016/0360-3016(94)00653-3

[43] Laughlin KM, Evans SM, Jenkins WT, Tracy M, Chan CY, et al (1996) Biodistribution of the nitroimidazole EF5 (2-[2-nitro-1H-imidazol-1-yl]-N-(2,2,3,3,3-pentafluoropropyl) acetamide) in mice bearing subcutaneous EMT6 tumors. J Pharmacol Exp Ther 277: 1049–1057. 8627516

[44] Dreher MR, Liu W, Michelich CR, Dewhirst MW, Yuan F, et al (2006) Tumor vascular permeability, accumulation, and penetration of macromolecular drug carriers. J Natl Cancer Inst 98: 335–344. 16507830 10.1093/jnci/djj070

[45] Potiron VA, Abderrhamani R, Giang E, Chiavassa S, Di Tomaso E, et al (2013) Radiosensitization of prostate cancer cells by the dual PI3K/mTOR inhibitor BEZ235 under normoxic and hypoxic conditions. Radiother Oncol 106: 138–146. 23321494 10.1016/j.radonc.2012.11.014

