# Supplementary figures and images for: Improved Functionality of the Vasculature during Conventionally Fractionated Radiation Therapy of Prostate Cancer
Source: PLoS One. 2013 Dec 31;8(12):e84076. doi: 10.1371/journal.pone.0084076 (PMC3877206; doi:10.1371/journal.pone.0084076)

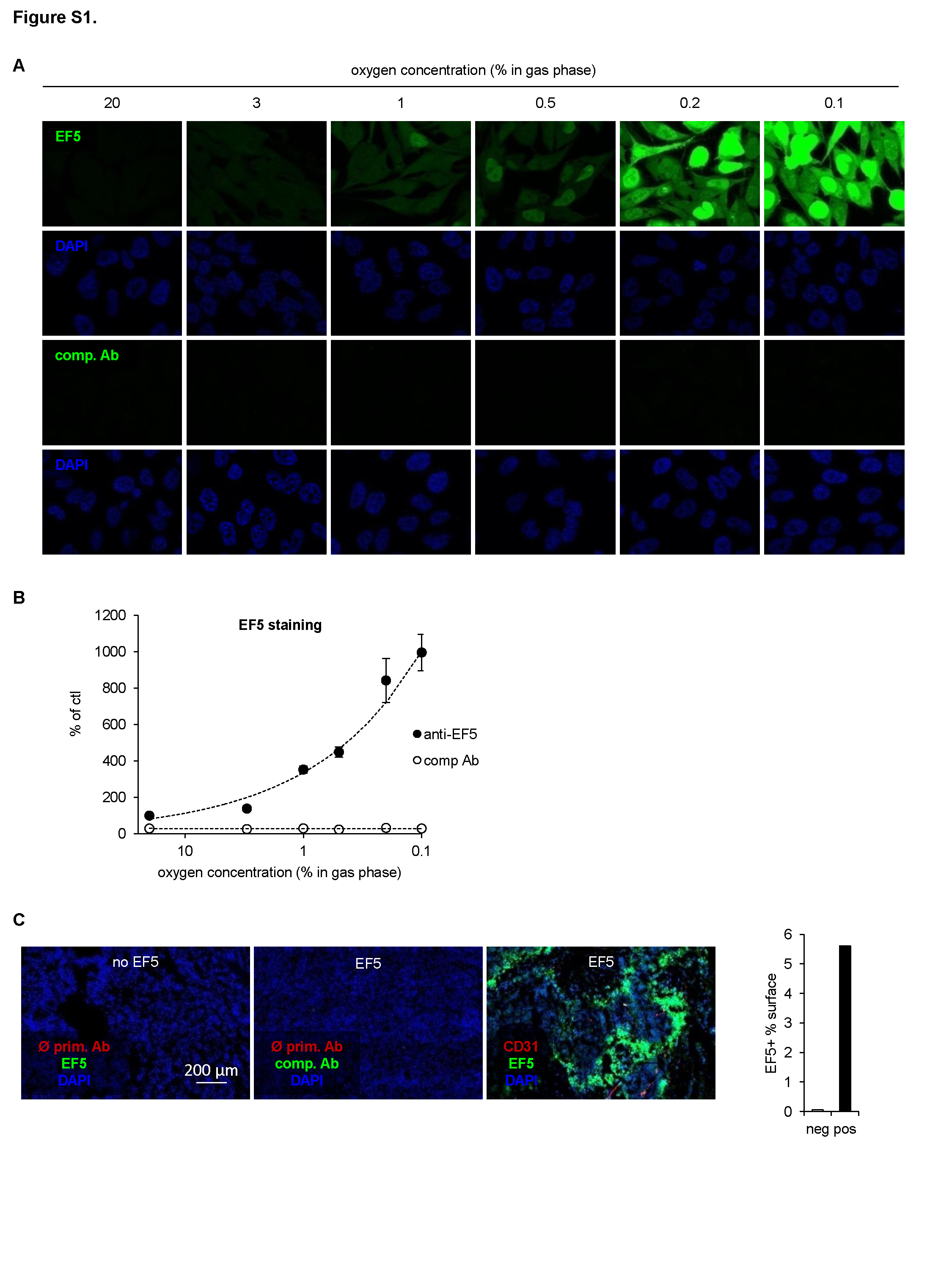

Supplement: Figure S1 — Oxygen sensitivity of EF5 in PC3 cells. (A) Images of cells cultured as indicated and exposed to EF5 for 2h and stained using anti-EF5 (top) or competed antibody (bottom). (B) Image quantification of EF5 staining observed in (A). Values represent the average of n = 3 per point ± sem and are normalized to 21% O2 (value = 100). (C) Pseudo-confocal images of tumors injected (middle and right) or not (left) with EF5 and stained using anti-EF5 (left, right) or competed antibody (middle). Graph: EF5+ surface in uninjected ("neg") and injected ("pos") hypoxic tumor. (TIF) [file pone.0084076.s001.tif]

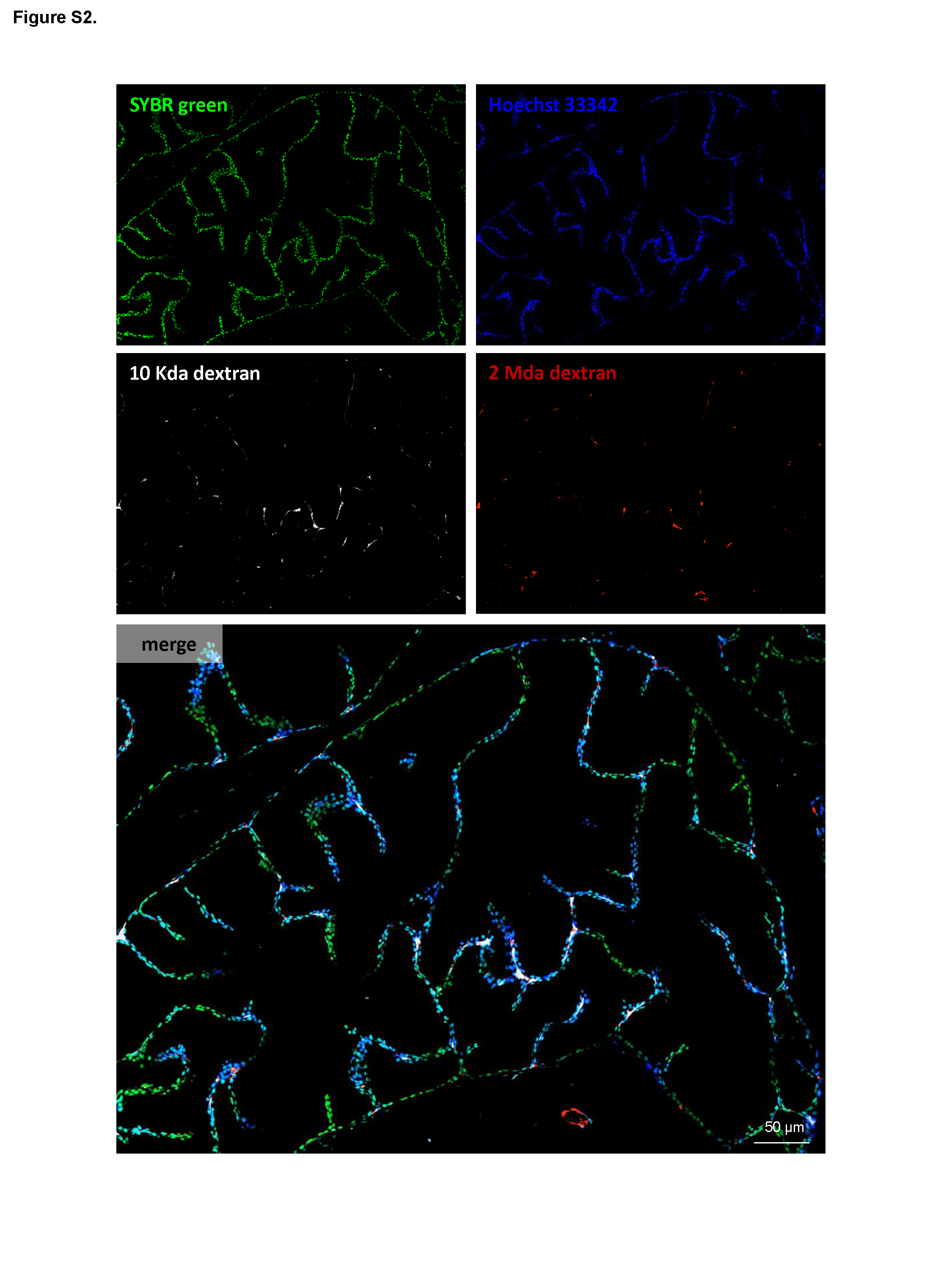

Supplement: Figure S2 — Perfusion of the normal prostate. Pseudo-confocal images of the normal prostate of untreated mouse injected with Hoechst 33342 and 10 kDa/2 MDa dextrans. SYBR green was used as a counterstain of total cell nuclei. (TIF) [file pone.0084076.s002.tif]

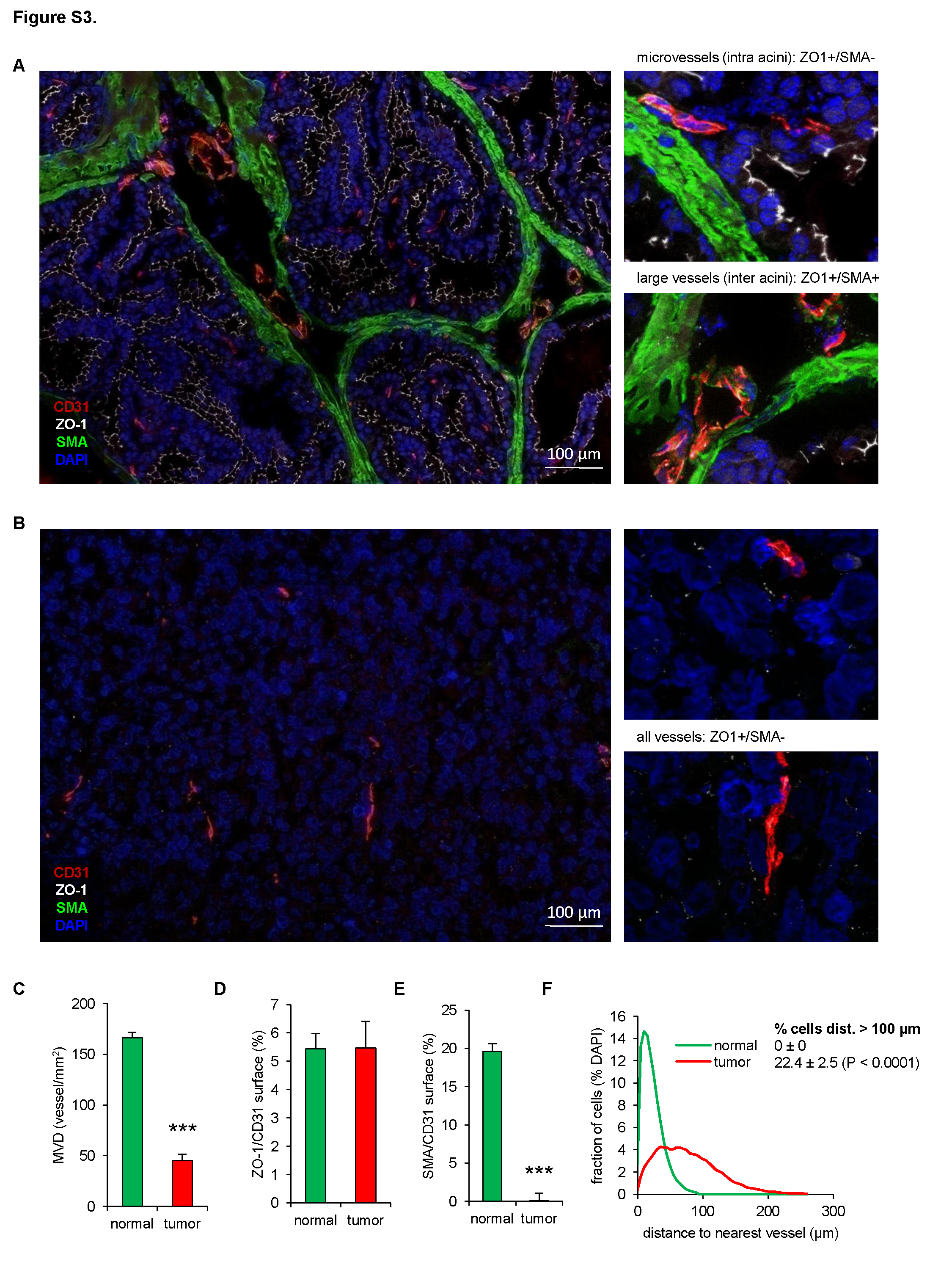

Supplement: Figure S3 — Normal and tumor-associated vasculature of the prostate. (A) Pseudo-confocal images of the normal prostate of untreated mouse stained for CD31/ZO-1/α-SMA. (B) Pseudo-confocal images of untreated mouse prostate tumor stained for CD31/ZO-1/α-SMA. (C,D,E) Quantifications in untreated normal mouse prostate ("normal") and untreated mouse prostate tumor ("tumor"). (F) Distance profile between cells and the closest blood vessel in untreated normal and tumor mouse prostate. Profiles are based on n≥6. (C,D,E,F). Statistical comparisons vs. normal. (TIF) [file pone.0084076.s003.tif]

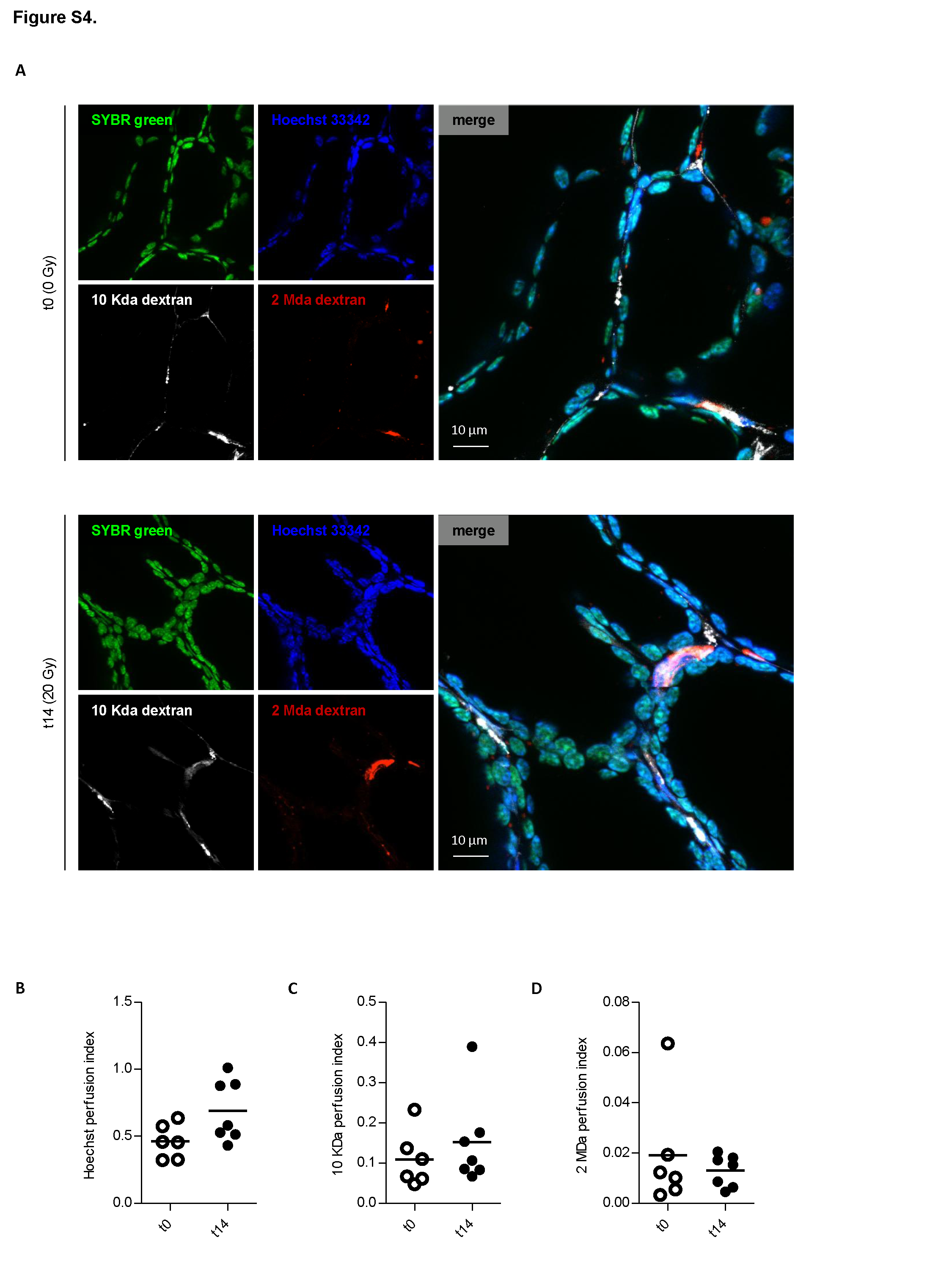

Supplement: Figure S4 — Fractionated irradiation does not increase perfusion of normal prostate acini. (A) Pseudo-confocal images of normal prostate acini perfused with Hoechst 33342 and 10 kDa/2 MDa dextrans before (t0) or after 2 weeks of CFRT (t14). SYBR green was used as a counterstain of total cell nuclei. (B,C,D) Image quantification of Hoechst+ (B), and medium (C) and large (D) dextran+ surfaces in normal prostate acini during CFRT (n = 6). Statistical comparisons vs. t0. (TIF) [file pone.0084076.s004.tif]

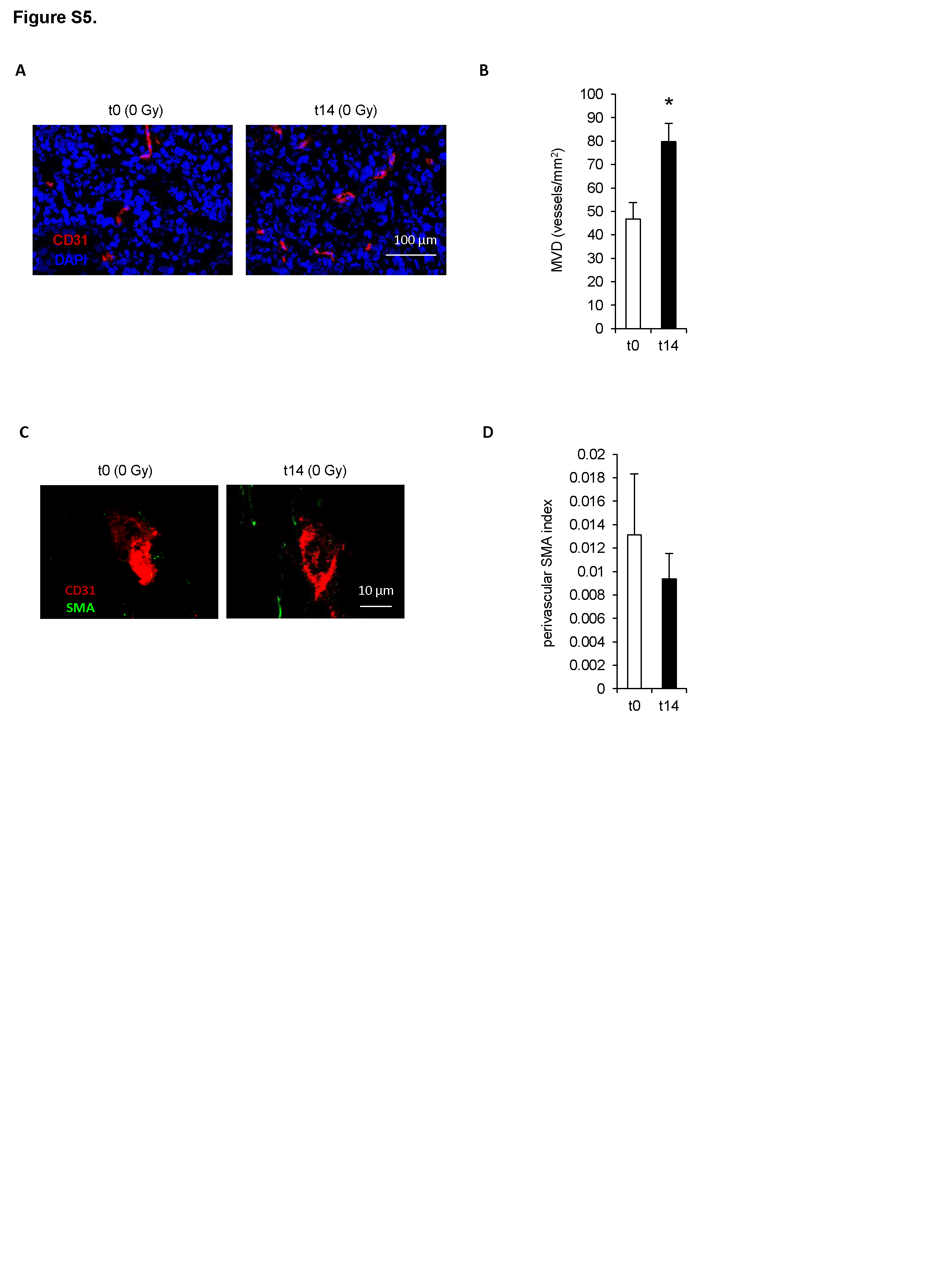

Supplement: Figure S5 — Non-irradiated tumors exhibit increased MVD but not vascular maturation. (A, B) Microvessel density in sham-irradiated (0 Gy) tumors. (A) Pseudo-confocal images. (B) Quantification; values represent the average of n≥13 per point ± sem. (C) Pseudo-confocal images of non-irradiated tumor blood vessels stained for SMA/CD31. (D) Image quantification of peri-CD31+ α-SMA surface. Values represent the average of n≥13 per point ± sem. (TIF) [file pone.0084076.s005.tif]

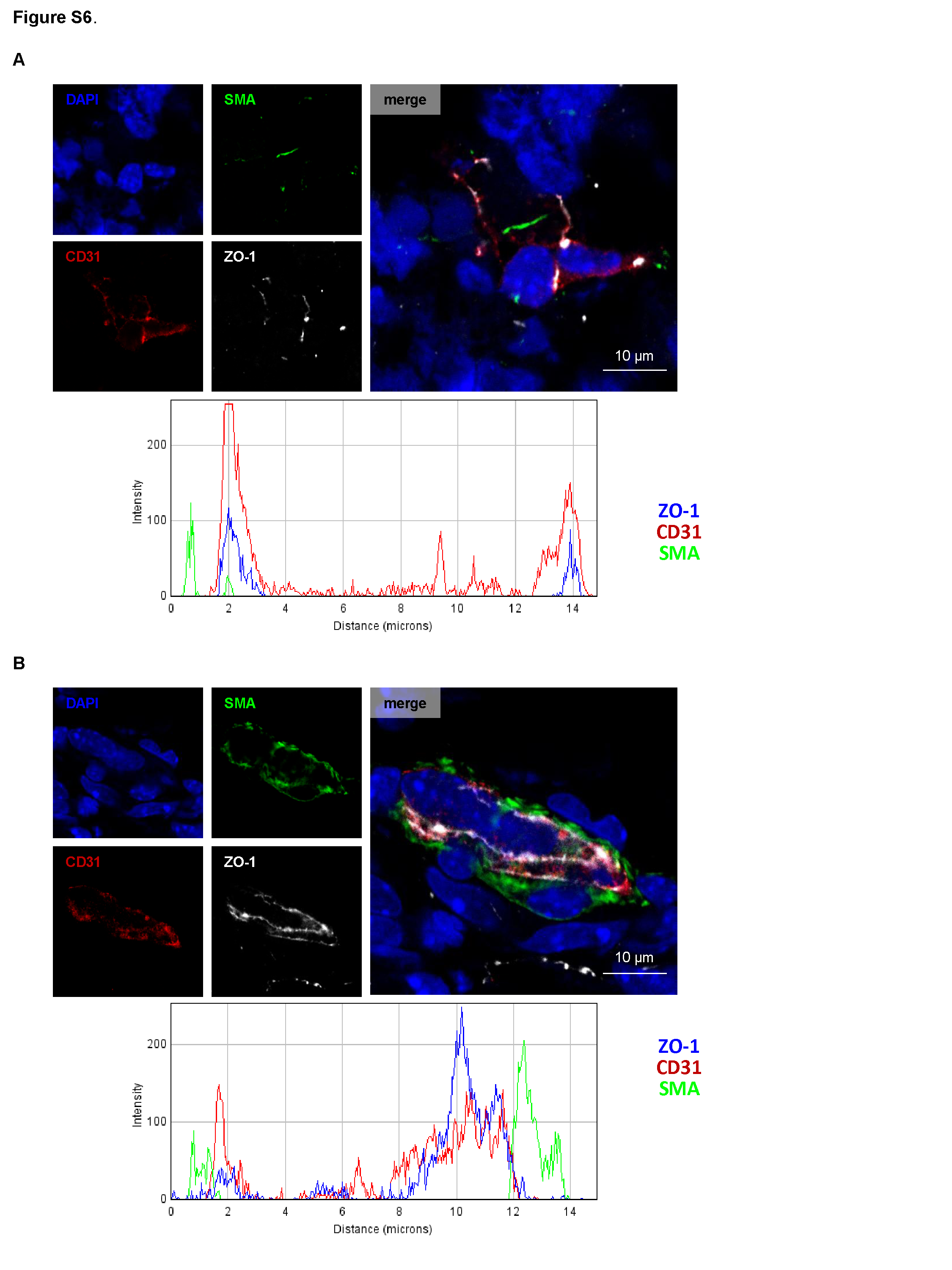

Supplement: Figure S6 — Endothelial distribution of ZO-1 and perivascular distribution of SMA. (A,B) Top: Representative confocal images of a blood vessel from an untreated (t0, A) or a 2-week treated (t14, B) tumor stained for CD31/ZO-1/SMA. Bottom: Histogram analysis of CD31/ZO-1/SMA pseudocolor profile of confocal image cross-section from (A or B). (TIF) [file pone.0084076.s006.tif]

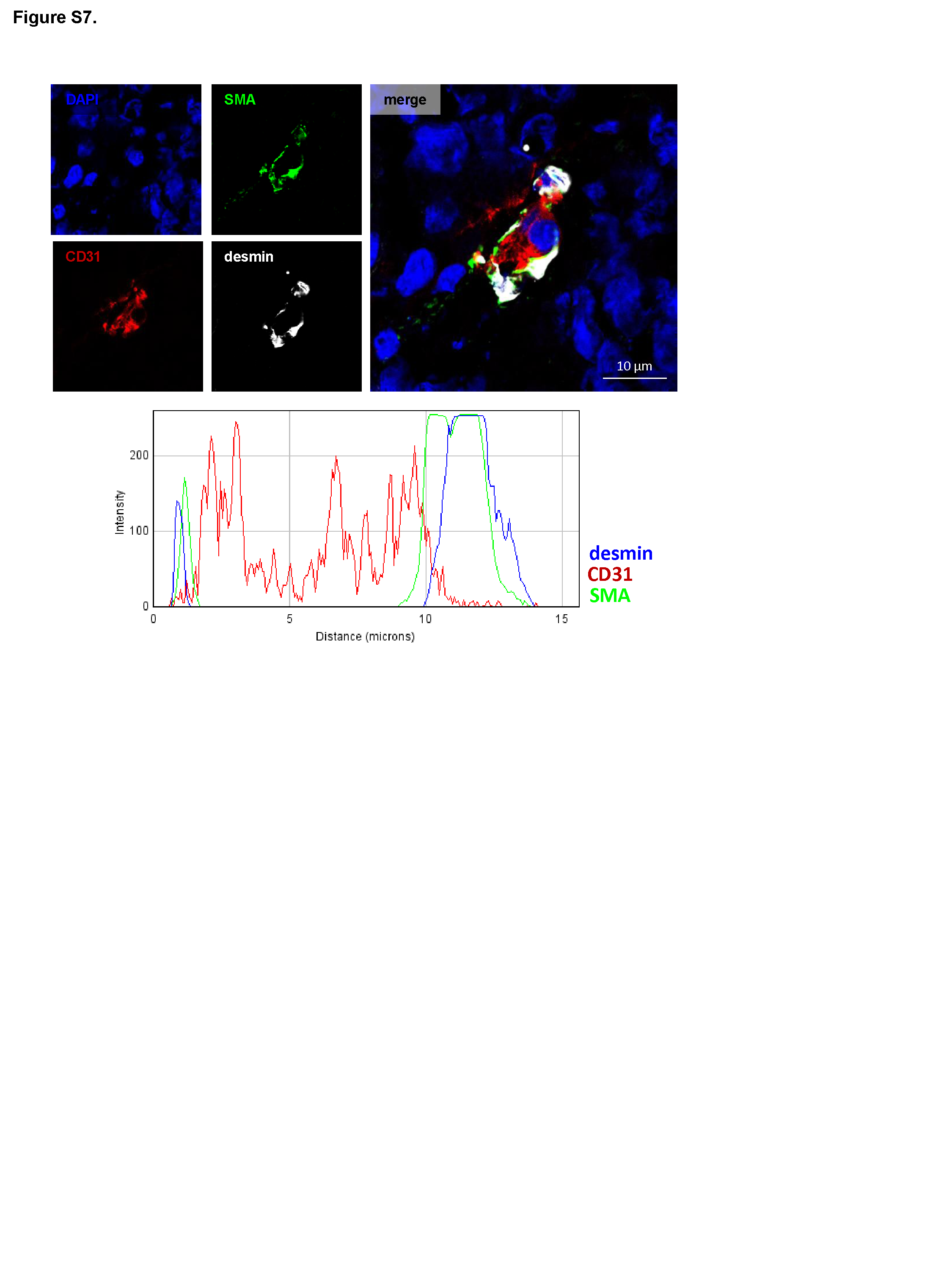

Supplement: Figure S7 — Perivascular co-expression of desmin and SMA. Top: Representative confocal images of a blood vessel from an untreated (t0) tumor stained for CD31/desmin/SMA. Bottom: Histogram analysis of CD31/desmin/SMA pseudocolor profile of confocal image cross-section. (TIF) [file pone.0084076.s007.tif]

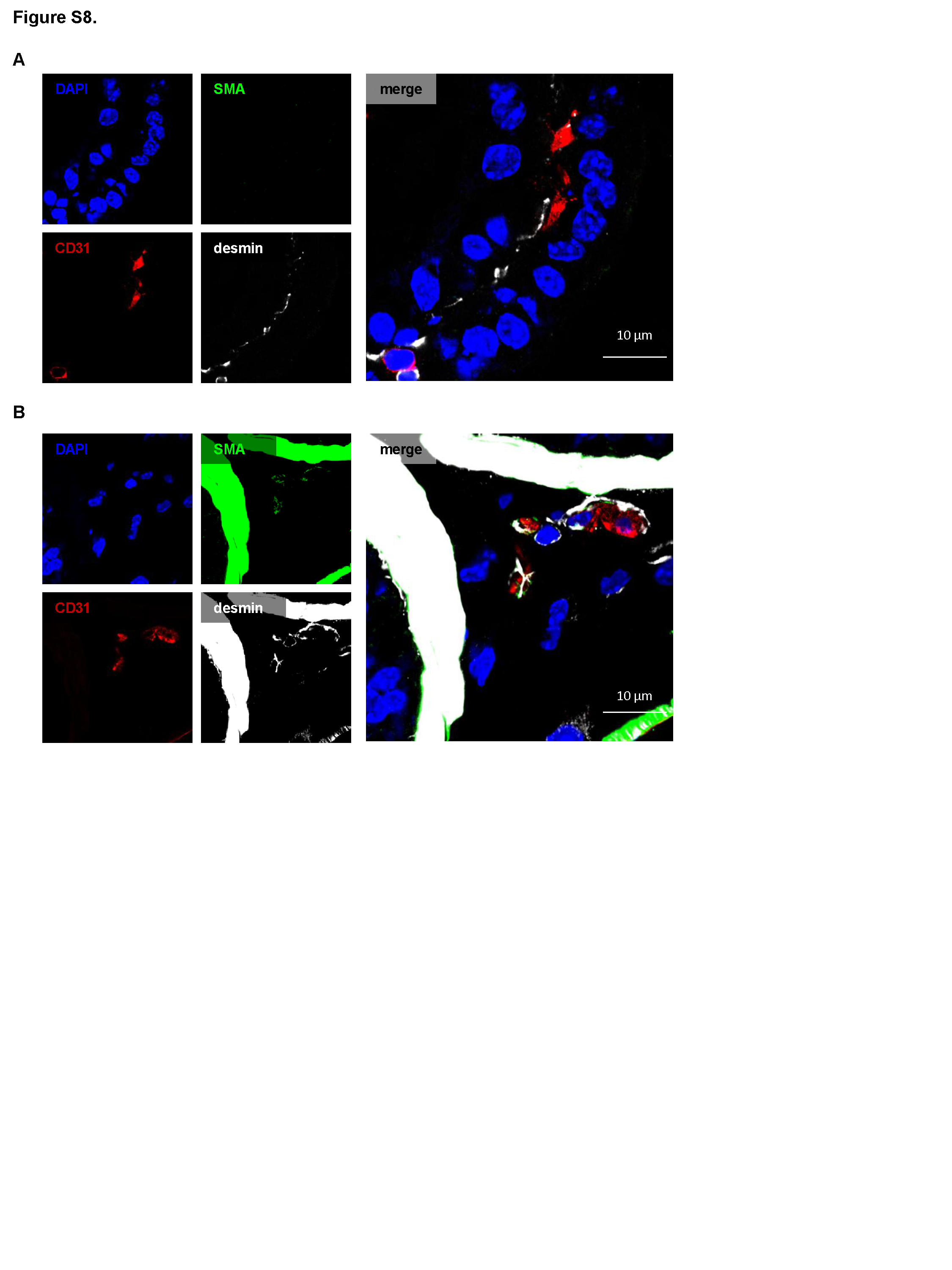

Supplement: Figure S8 — Co-expression of desmin and SMA in the normal prostate. (A,B). Representative confocal images of a blood vessel from an untreated normal mouse prostate stained for CD31/desmin/SMA. (A) intra- and (B) inter-acinus region. (TIF) [file pone.0084076.s008.tif]

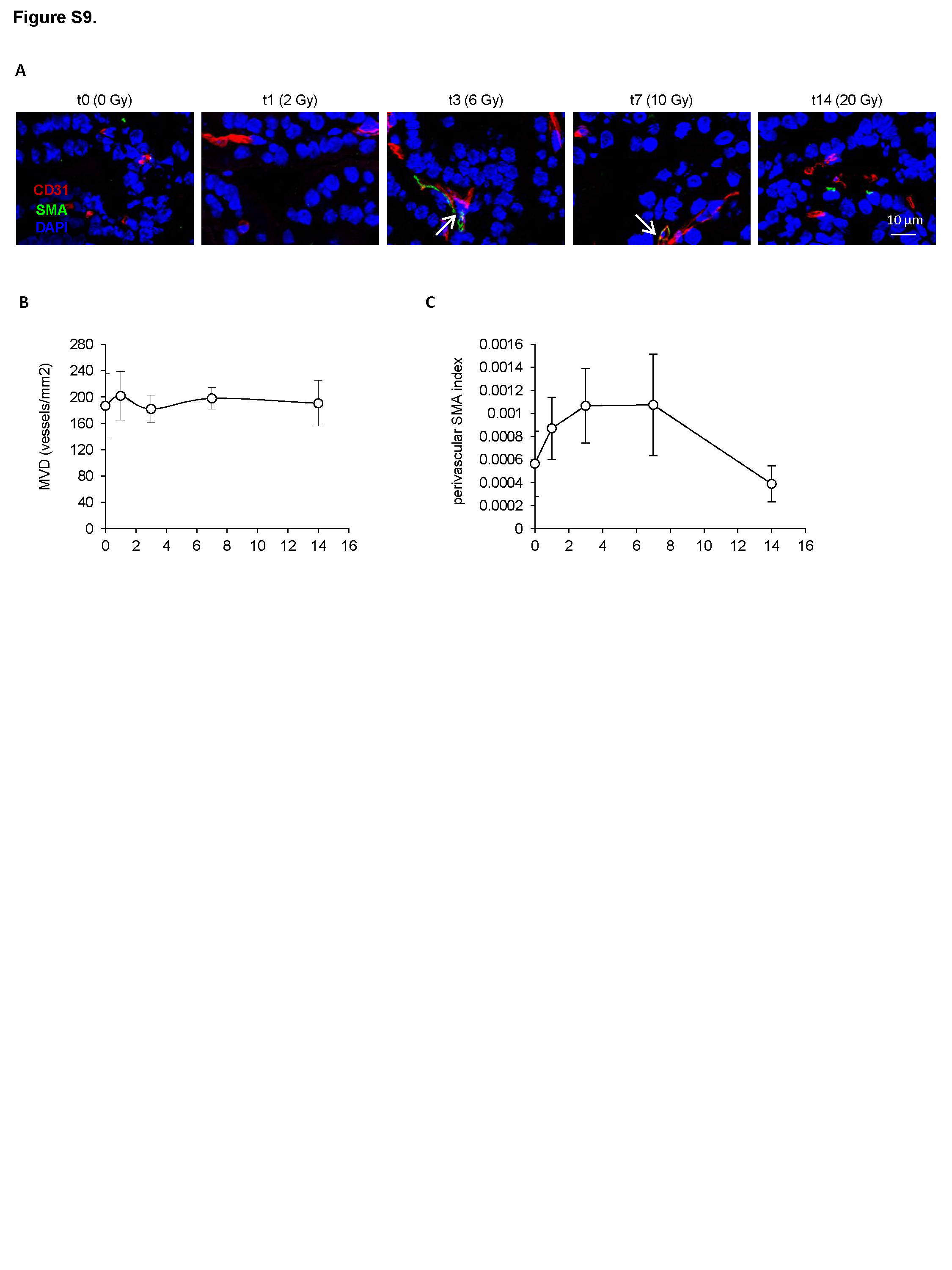

Supplement: Figure S9 — Irradiated microvessels of normal prostate acini exhibit no significant changes in MVD or vascular maturation. (A) Pseudo-confocal images of normal blood vessels stained for SMA/CD31 during CFRT. (B) Microvessel density of normal prostate acini during CFRT. Values represent the average of n≥13 per point ± sem. (C) Image quantification of peri-CD31+ SMA surface. Values represent the average of n≥13 per point ± sem. (TIF) [file pone.0084076.s009.tif]
